# Arbuscular mycorrhizal fungi increased peanut (*Arachis hypogaea* L.) yield by changing the rhizosphere microbial community structure in saline-alkali soil

**DOI:** 10.3389/fmicb.2023.1303979

**Published:** 2023-12-08

**Authors:** Jia Kang, Wenlong Yang, Shangwu Liu, Ning Xuan, Yahui Shao, Yun Geng, Muhammad Afzal, Yingxin Zhang, Shousong Yue, Rubina Mushtaq, Gao Chen

**Affiliations:** ^1^Institute of Crop Germplasm Resources, Shandong Academy of Agricultural Sciences, Jinan, China; ^2^State Key Laboratory of Nutrient Use and Management, Jinan, China; ^3^Economic Crop Research Institute Heilongjiang Academy of Agricultural Sciences, Harbin, China; ^4^Institute of Molecular Biology and Biotechnology, The University of Lahore, Lahore, Pakistan

**Keywords:** arbuscular mycorrhizal fungi, saline-alkali soil, peanut, soil microorganisms, soil enzyme

## Abstract

Arbuscular mycorrhizal fungi (AMF) have demonstrated the potential to enhance the saline-alkali tolerance in plants. Nevertheless, the extent to which AMF can ameliorate the tolerance of salt-sensitive plants to alkaline conditions necessitates further investigation. The current study is primarily centered on elucidating the impact of AMF on the growth of the Huayu22 (H22) when cultivated in saline-alkaline soil. We leveraged DNA of rhizosphere microorganisms extracted from saline-alkali soil subjected to AMF treatment and conducted high-throughput sequencing encompassing 16S rRNA gene and ITS sequencing. Our findings from high-throughput sequencing unveiled Proteobacteria and *Bacillus* as the prevailing phylum and genus within the bacterial population, respectively. Likewise, the predominant fungal phylum and genus were identified as Ascomycota and *Haematonectria*. It is noteworthy that the relative abundance of Proteobacteria, Actinobacteria, Chloroflexi, Bacteroidetes, and Ascomycota exhibited significant increments subsequent to AMF inoculation. Our investigation into soil enzyme activity revealed a remarkable surge post-AMF inoculation. Notably, the amounts of pathogen growth inhibitory enzymes and organic carbon degrading enzymes rise, as predicted by the putative roles of microbial communities. In saline-alkali soil, inoculation of AMF did boost the yield of H22. Notable improvements were observed in the weight of both 100 fruits and 100 grains, which increased by 20.02% and 22.30%, respectively. Conclusively, this study not only provides a theoretical framework but also furnishes empirical evidence supporting the utilization of AMF as a viable strategy for augmenting the yield of salt-sensitive plants grown in alkaline conditions.

## 1 Introduction

In the semi-arid tropics (SAT), which include parts of Africa, Asia, North America, and South America, where extremes of drought and soil salinity are common, approximately 60% of the world’s peanut crop is produced (Mace et al., 2006; Cuc et al., 2008). Unfavorable environmental factors significantly impact peanut output and growth. High salt concentrations also lead to physiological dryness and ion toxicity, inhibit peanut growth, biomass, yield, photosynthesis, and water use efficiency (Chao et al., 2022). Consequently, peanut roots, stems, and leaves are unable to develop properly. To address the shortage of cultivable land and promote regional agriculture, attempts have been made to cultivate peanuts in saline-alkaline soil in northern China, especially in the coastal regions of the Yellow River Delta (Ci et al., 2021). However, since peanuts are particularly sensitive to salinity, salinity stress interferes with their growth at various stages, including seed germination, chlorophyll production, pod development, and fodder production, ultimately reducing crop yield (Avishek et al., 2018). Being a saline-alkali-sensitive plant, H22 production and quality suffer due to the impact of saline-alkali soil. Consequently, one of the main breeding objectives in the peanut industry and saline-alkali land usage projects is to further improve peanut salt tolerance.

Worldwide, soil salinity is becoming a significant problem as it is encountered in all climates, seriously restricting crop yields and plant growth (Evelin et al., 2019). Approximately 9 × 10^8^ m^2^ of land, or 25% of the world’s surface area, is affected by salinization. According to research, 30% of cultivated land will convert into saline soil within 20 years, and this figure may rise to 50% within 30 years. This will have an increasingly negative impact on plant growth and the ecological environment worldwide (Porcel et al., 2012). Recent years have seen an increase in soil salinity due to over-cultivation and growing industrialization, leading to secondary soil salinization. One of the most significant environmental problems affecting humanity is salinization. Biodiversity is significantly impacted by salinization, and saline habitats require more time to restore than other soil ecosystems (Bless et al., 2018). High salt concentrations cause physiological dryness and ion toxicity, in addition to limiting plant growth, biomass, yield, photosynthesis, and water usage efficiency. High salinity forces plants to absorb more harmful ions like Na^+^ and Cl^−^ while dramatically reducing their ability to absorb phosphorus and potassium (Khan et al., 2019). Plant roots, stems, and leaves grow and develop at a much slower rate. As a result, improving saline soil and increasing plant salt tolerance are currently key priorities.

Important plant activities, such as nitrogen fixation and phosphorus solubilization depend heavily on the rhizosphere microbiome (Ceja-Navarro et al., 2021). On the other hand, root-associated microbiota carried out specific functions, including element metabolism and transformation (for instance, nitrogen and phosphate cycling), which are beneficial for the growth of their host and affect plant fitness (Huang et al., 2022). The bacterial community structure associated with peanuts may be unique, while changes in composition and their relationships with salt stress and peanut cultivars remain unknown. Soil pollution contributes uncertain effects and may cause different shifts in the root-associated microbiome of various niches (e.g., bulk soil, rhizosphere, rhizoplane, and endosphere), which need to be clarified. The application of arbuscular mycorrhizal fungi (AMF) is one of the most significant, environment friendly, and economically advantageous bioremediation technique for managing abiotic stress in soil and plants (Dhalaria et al., 2020). The various metabolic abilities of rhizosphere-associated microorganisms enable them to actively influence plant growth and tolerance to biotic and abiotic stresses (Dennis et al., 2010). Most plants can establish a healthy symbiotic connection with AMF, one of the significant soil microbes, which enhances plant water metabolism and salt tolerance (Wang et al., 2019).

Most of the research on arbuscular mycorrhizal fungi (AMF) conducted thus far has focused on how AMF inoculation affects plant performance or the general composition and functioning of microbial communities in pots or agricultural soil (Elliott et al., 2021; Basiru and Hijri, 2022). However, the effects of AMF application on salt-sensitive plants and the microbial population in saline-alkaline soils are less well understood. Given the wide-ranging impact of the soil microbiome on plant performance, it is crucial to investigate how AMF affects the microorganisms associated with peanut roots and how this correlates with crop growth in order to increase peanut yields in saline-alkaline soil. Our research encompassed an examination of root-associated microorganisms, soil enzyme activity, peanut growth, and pod yield, with the aim of shedding light on the intricate interplay between AMF and these critical components in saline-alkaline soil conditions.

## 2 Materials and methods

### 2.1 Site description

The experiment was conducted at Dongying Experimental Station in China (longitude 118° 39′ 36″ E, latitude 37° 19′ 12″ N). The field had been under rotation cultivation of peanut, wheat (*Triticum aestivum*), and maize (*Zea mays*) for more than 5 years. The study employed the saline and alkali-sensitive peanut variety H22, which was preserved by the Institute of Crop Germplasm Resources, Shandong Academy of Agricultural Sciences. The field soil was air-dried, ground, and sieved through a 2 mm mesh for physicochemical analysis. The soil’s physicochemical properties were as follows: soil organic matter (SOM) 14.56 g·kg^−1^, total nitrogen (TN) 1.16 g·kg^−1^, total phosphorus (P_2_O_5_) 0.91 g·kg^−1^, microbial carbon 196.60 mg·kg^−1^, NaCl 2.50 g·kg^−1^, available nitrogen 40.40 mg·kg^−1^, and available phosphorus 39.60 mg·kg^−1^.

### 2.2 Peanut inoculation and planting

Two groups of plants were examined: the control group (without AMF treatment) and the experimental group (with AMF treatment). In the AMF treatment group, the seeds were inoculated with AMF (*Rhizophagus irregularis* SA: *Funneliformis mosseae* BEG95 = 1:1) using a seed coating agent (0.1 kg AMF was inoculated onto 1 kg of seeds). The AMF agent was placed 10 cm below the soil surface and sown in soil containing a 1:1.5:1.5 (N-P_2_O_5_-K_2_O) fertilizer (975 kg·ha^−1^). The AMF were provided by Symbiom Ltd. in powder form, with 252 spores g^−1^ used for seed treatment (certified by Symbiom Limited company).

To ensure thorough moisture saturation, the field was plowed one day before sowing and received two rounds of irrigation. The treatment seeds were manually planted in seeding holes within raised beds (10 cm high × 85 cm wide). After emergence, the seedlings were thinned to two plants per hole. Each plot consisted of 10 rows with a 50 cm gap between rows. Each treatment was replicated three times. The plants were irrigated twice per week with 25 mm of water.

### 2.3 Sample collection of bulk soil, ectorhizosphere, rhizosphere, rhizoplane, and endosphere fractions

**Figure 1:**
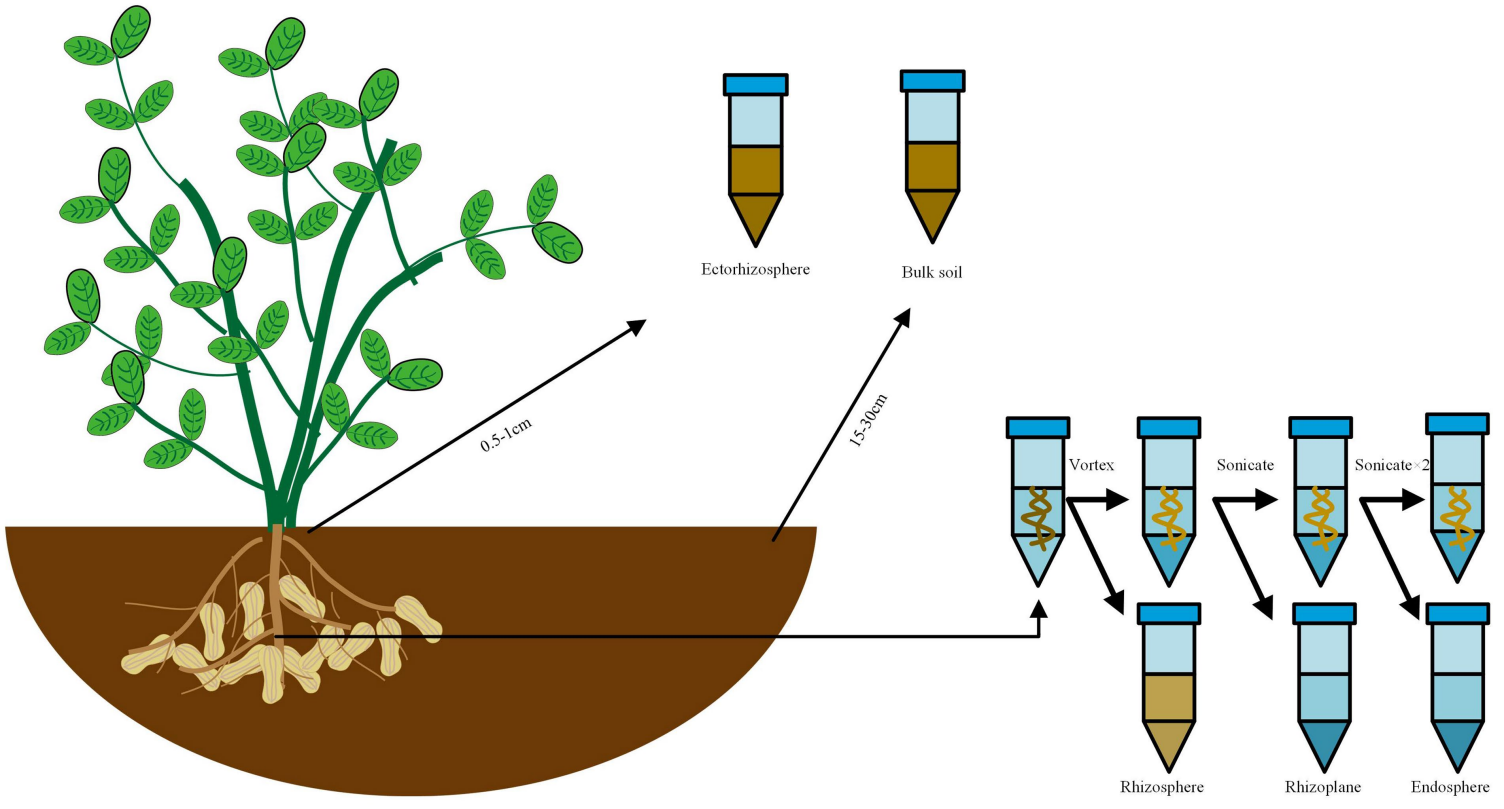
Sampling and collection of the rhizocompartments.

All samples were obtained in five replicates. The sample collection methodology was refined based on Edwards’ method (Edwards et al., 2015). The sampling method was shown in Figure 1. Bulk soil samples: the samples were obtained approximately 5 cm below the soil surface and positioned 15–30 cm away from the plant, then placed in 15 mL tubes and kept at 4°C until DNA extraction on the same day (The control group was named as “RO,” while the experimental group as “ROA”). Ectorhizosphere soil samples: the samples were collected 0.5–1 cm away from the root, then placed in 15 mL tubes and kept at 4°C until DNA extraction on the same day (The control group was named as “RA,” while the experimental group as “RAA”). Rhizosphere soil samples: the samples were obtained from approximately 1 mm layer of soil around the roots, then placed in 50 mL tubes containing sterile Phosphate Buffered Saline (PBS) solution and vigorously agitated to separate the soil from the roots. The roots were taken out for the extraction of rhizoplane samples, and the liquid PBS fraction was kept at 4°C until DNA extraction on the same day (The control group was named as “RR,” while the experimental group as “RRA”). Rhizoplane samples: the roots designated for rhizoplane collection were sonicated for 30 s at 50–60 Hz (output frequency 40 kHz, power 480 W, Branson Scientz) in 15 mL PBS. The roots were then removed for the extraction of endosphere samples, and the liquid PBS fraction was kept at 4°C until DNA extraction on the same day (The control group was named as “RS,” while the experimental group as “RSA”). Endosphere samples: the roots designated for endosphere collection were sonicated two times in PBS and stored at −80°C until DNA extraction on the same day (The control group was named as “RI,” while the experimental group as “RIA”).

### 2.4 DNA extraction from rhizocompartments

Total DNA was extracted from the samples by using FastDNA Spin Kit (MP Biomedicals, LLC., Solon, OH, USA) following the manufacturer’s instructions. A Nanodrop ND-2000 UV–vis spectrophotometer (NanoDrop Technologies, Wilmington, DE, USA) was used to measure DNA purity and quantity, while 1% agarose gel electrophoresis was used to observe DNA size and integrity. For high-throughput sequencing based on Illumina MiSeq, the Illumina HiSeq 2,500 platform (Illumina Inc., San Diego, CA, USA) was used to amplify PCR products from the purified DNA samples and to sequence 16S rRNA gene and ITS1 (internal transcribed spacer) regions.

Total DNA from 50 samples (5 compartments × 2 treatments × 5 replicates/treatment) was analyzed to examine 16S rRNA gene regions V3-V4 for the bacterial community and ITS region ITS1 for the fungal community. FLASH was used to merge the paired-end reads from original DNA fragments (Magoc and Salzberg, 2011). Paired-end reads were assigned to each sample based on their unique barcodes. The sequences were clustered at 97% similarity using the UPARSE algorithm (Edgar, 2013), generating operational taxonomy units (OTUs). To assign taxonomies to these OTUs, we used the EzBioCloud database (Yoon et al., 2017) for 16S rRNA gene and Unite (Kõljalg et al., 2005) for ITS1. The dilution curve showed that the gene curve of bacterial 16S rRNA gene tended to be stable at 20,000 sequences (Supplementary Figure S1A), while the gene curve of fungal ITS1 tended to be stable at 15,000 sequences (Supplementary Figure S1B), indicating that the number of sequences was saturated and that the current sequencing protocol accurately reflects the composition of the sample. To generate a maximum likelihood tree, we used MUSCLE to align the representative sequences of each OTU and FastTree to construct the phylogenies. The USEARCH (Edgar, 2010), and QIIME (Kuczynski et al., 2012) platforms were used jointly to perform the above steps. The functional potentials of the bacterial and fungal communities in soil samples were predicted via phylogenetic investigation of the communities by reconstructing the unobserved states pipeline using PICRUSt v1.1.2 (Langille et al., 2013). Functional profiling was conducted using the Kyoto Encyclopedia of Genes and Genomes (KEGG) database.

### 2.5 Enzymatic activities analyses

According to Guan (1986), we measured the activities of catalase, urease, phosphatase, and cellulase. Catalase activity was determined by back-titrating residual H_2_O_2_ with a standard KMnO_4_ solution. Urease activity was measured using indophenol blue colorimetry. Phosphatase activity was assessed using the disodium phenyl phosphate colorimetric technique, and soil cellulase activity was determined using the DNS technique. The production of 1 μmol H_2_O_2_ per hour of uric acid decomposition per gram of sample is defined as an enzyme activity unit.

### 2.6 Yield parameters of peanut

To evaluate the influence of AMF on peanut growth, ten peanut plants from each experimental group were randomly selected when they had reached maturity. All samples were oven-dried to a consistent weight for 30 min at 105°C, followed by an additional 15 min at 80°C. The weights of 100-pods from the treatment and control groups were noted in order to ascertain the impact of AMF treatment on peanut yield.

### 2.7 Statistical analysis

Mothur (version v.1.30) was used to determine the alpha diversity indices, including Shannon diversity, Chao1, Simpson, and Goods coverage. R (v. 3.4.2) was used for correlation analysis, data presentation, and statistical analysis of 16S rRNA gene and ITS1. β diversity analysis was performed to examine similarities and differences between different soil groups, including principal component analysis (PCA), unweighted pair group method with arithmetic mean (UPGMA), and non-metric multidimensional scaling (NMDS). Variations in the microbial community across the two treatments were evaluated by PCA. Ggplot2 was used to prepare the graphs. These steps were performed using the R platform with the vegan and ggplot2 packages (David and Hadley, 2013). SourceTracker (v.1.0) based on the Bayesian approach (Knights et al., 2011) was used to estimate the sources of the RR, RS, and RI bacterial communities in each compartment.

## 3 Results

### 3.1 Diversity of the microbial community

In order to examine the impact of AMF on the composition of rhizosphere-related microbial communities in H22 under salt stress, both the inoculated and uninoculated AMF were sequenced. A total of 4,249,405 high-quality fungal sequences and 4,218,906 high-quality bacterial sequences were recovered, ranging from 63,633 to 81,686 sequences per sample for the former and 53,550 to 85,725 sequences for the latter. Bacteria and fungi had coverage rates of 98.59% and 100.00%, respectively. Clustering techniques yielded 1,852 bacterial and 835 fungal OTUs from all samples (Supplementary Figures S1A,C). After the removal of doubtful sequences, 3,674,807 bacterial sequences and 3,986,806 fungal sequences passed quality screening. Most of the bacterial sequence lengths were found to be between 400 and 500 bp, while the fungal sequence lengths were found to be between 200 and 300 bp. Subsequently, high-quality reads were grouped into operational taxonomic units (OTUs) using a standard threshold of >97% sequence identity. The complete lists of OTUs for each soil group are displayed in Supplementary Figures S1B,D, with RI having the fewest and RO having the greatest number of OTUs.

Alpha diversity analysis was conducted to investigate the community richness and diversity of bacterial and fungal ecologies in each sample connected to the peanut rhizosphere. Rarefaction curve analysis indicated that each sample had a high sequencing depth, and the sequencing depth of all samples was sufficient for determining the composition of the microbial community (Supplementary Figures S2A,C). The rank abundance curve demonstrated significant species evenness and homogeneity in RS and RI (Supplementary Figures S2B,D).

**Figure 2:**
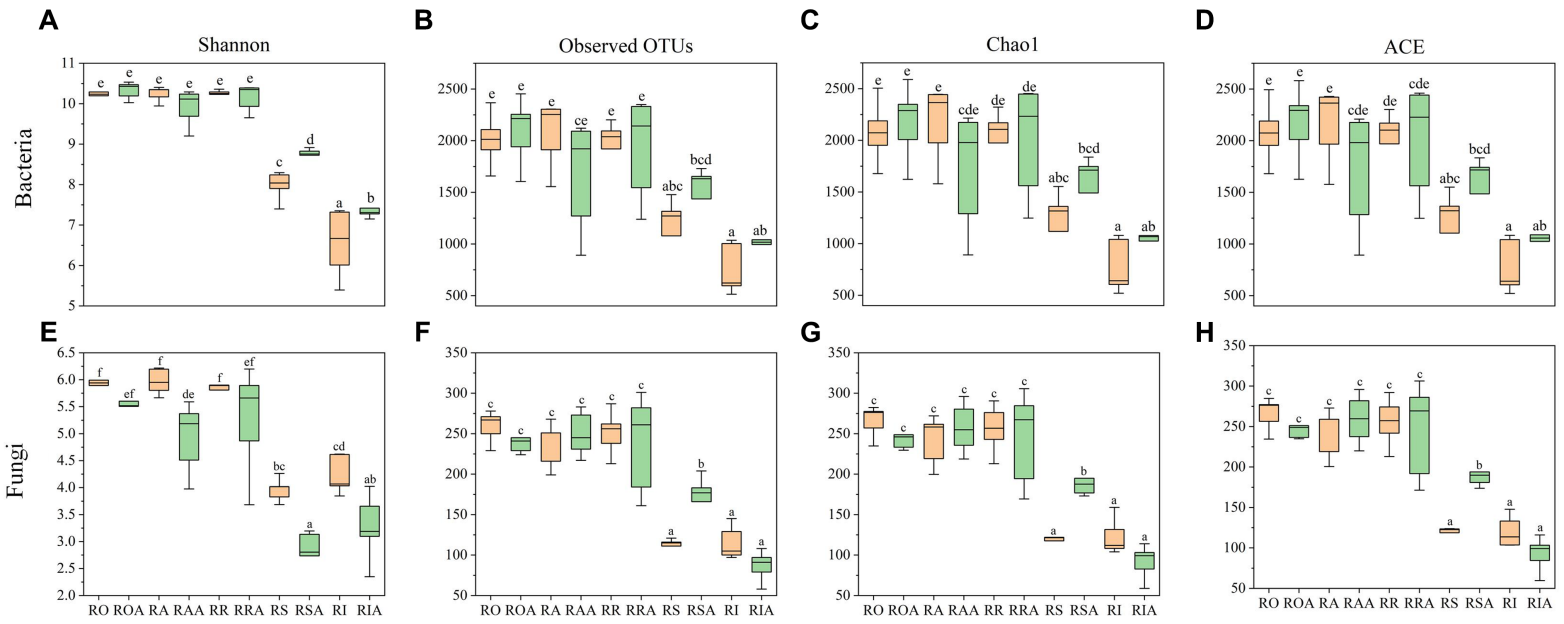
Microbial diversity of control group and AMF treatment. **(A)** OTUs, **(C)** Shannon, **(E)** Chao 1 and **(G)** ACE indices representing bacterial communities, respectively; represented by **(B)** OTUs, **(D)** Shannon, **(F)** Chao 1 and **(H)** ACE index of fungal community, respectively. RO (bulk soil without AMF treatment); ROA (bulk soil with AMF treatment); RA (ectorhizosphere soil without AMF treatment); RAA (ectorhizosphere soil with AMF treatment); RR (rhizosphere soil without AMF treatment); RRA (rhizosphere soil with AMF treatment); RS (rhizoplane without AMF treatment); RSA (rhizoplane with AMF treatment); RI (endosphere without AMF treatment) and RIA (endosphere with AMF treatment).

In addition to Shannon, Simpson, and ACE, this study employed several other indices to represent the alpha diversity of the bacterial community (Figure 2). Among the control group samples, RS showed a significant difference from RO, RA, RR, and RA, while there was a difference, although not statistically significant, between RO, RA, RR, and RA. However, after AMF inoculation, noticeable variations were observed between ROA, RAA, RRA, RSA, and RAA. Furthermore, following AMF inoculation, diversity within the same root portions changed, particularly in bulk soil, rhizoplane, and endosphere. The bacterial community’s Shannon index increased, whereas the fungal community’s Shannon index decreased. Only the chao1 and ACE indexes of RAA decreased in the bacterial community, while they increased for the other root components. The fungal community’s ROA and RIA chao1 and ACE indexes decreased, while those for other root sections increased. In conclusion, AMF inoculation can alter the diversity of microbial communities associated with H22 roots.

### 3.2 Microbial community abundance

The Venn diagram (Supplementary Figure S3) illustrates the operational taxonomic categories before and after AMF inoculation. However, the bacterial community in the RO, RR, RA, RS, and RI treatment groups had 1,272, 1,150, 1,298, 694, and 899 OTUs, respectively, which were significantly lower in number compared to the ROA, RRA, RAA, RSA, and RIA treatment groups. The pattern of change in the fungus community was opposite to that of bacteria, except for RSA. These findings demonstrate that AMF altered the diversity of soil bacteria and promoted the proliferation of soil microorganisms in saline-alkaline soil, consistent with the results in Figure 2.

In all compartments, the 16S rRNA gene analysis identified 41 bacterial phyla, 523 families, and 1,679 genera. Among the bacterial communities, the most prevalent phyla in all samples were Proteobacteria (34.65%), Acidobacteriota (13.62%), Actinobacteriota (9.06%), Gemmatimonadetes (7.84%), Firmicutes (7.79%), and Planctomycetes (7.66%); 80% or more of all sequences. The top 10 most abundant phyla also included Chloroflexi (5.06%), Verrucomicrobia (3.78%), Bacteroidetes (3.68%), and Cyanobacteria (3.14%) (Supplementary Table 1). AMF inoculation markedly enhanced the relative abundance of Proteobacteria (34.65% *VS* 36.08%), Actinobacteriota (9.06% *VS* 11.79%), Chloroflexi (5.06% *VS* 5.49%), and Bacteroidetes (3.68% VS 3.89%) among the top 10 phyla. Conversely, AMF treatment reduced the relative abundance of Acidobacteriota, Gemmatimonadetes, Firmicutes, Planctomycetes, Verrucomicrobia, and Cyanobacteria. The genus *Bacillus* was the most prevalent prevalent (6.76%). Additionally, although making up less than 20% of all sequences, the genera *Sphingomonas* (2.04%) and *Gemmatimonas* (1.30%) exhibited significant abundances (Supplementary Table 2). As a result, AMF inoculation altered the abundances of various bacterial groups, in addition to changes at the phylum and genus levels.

14 fungal phyla, 164 families, and 263 genera were identified throughout all compartments using ITS1 analysis. In all samples, the top 10 most dominant phyla in the fungal community were Ascomycota (70.92%), Zygomycota (7.79%), Basidiomycota (6.01%), Fungi unclassified (5.70%), Glomeromycota (5.54%), Chytridiomycota (1.83%), Mortierellomycota (0.41%), Mucoromycota (0.06%), and Entomophthoromycota (0.01%), accounting for more than 99.00% of the sequences. The relative abundances of the top 10 phyla were dramatically changed by the AMF treatment. For instance, Ascomycota saw a large rise in relative abundance following AMF inoculation, while the abundances of other prominent phyla declined (Supplementary Table 3). The most abundant fungal genera in all samples were *Haematonectri*a (14.15%), *Talaromyces* (8.40%), *Mortierella* (6.14%), *Gibellulopsis* (4.89%), *Metacordyceps* (4.66%), *Ascobolus* (3.39%), *Kotlabaea* (1.50%), *Neonectria* (2.09%), *Cladorrhinum* (2.20%) and *Gibberella* (2.52%), accounting for 50% of all sequences. The relative abundances of *Haematonectria* (14.15% *VS* 15.63%), *Talaromyces* (8.40% *VS* 14.42%), *Neonectria* (2.09% *VS* 3.16%) and *Kotlabaea* (1.50% *VS* 5.15%) among the top 10 genera increased significantly after AMF inoculation, but the relative abundances of the other prominent genera declined (Supplementary Table 4).

### 3.3 Comparison of species composition in peanut rhizosphere soil

To compare and contrast the various soil and root compartments, including PCA and UPGMA, we conducted a diversity analysis. By using PCA, we assessed differences in the microbial community between the two treatments. The total variability depending on OTU level was described by the first two primary components of the bacterial and fungal communities (Supplementary Figure S4).

The plots clearly showed differences between the two treatments in the bacterial and fungal communities of peanut in, on, and close to the root. Generally speaking, the five replicates for each treatment was grouped together and displayed separation from the other treatments. Greater separations across treatments along PC1 and PC2 suggest that AMF had a stronger impact on the community structure of bacteria than fungi. The five repeated samples in the same compartment tended to cluster, according to UPGMA, which also revealed that the bacterial community structure varied between compartments (Supplementary Figures S5A,B). AMF inoculation changed the abundance distribution of bacteria and fungi in the saline-alkaline soil hosting peanut, as evidenced by the considerable separation of bacterial and fungal community structures in various rhizosphere compartments.

**Figure 3:**
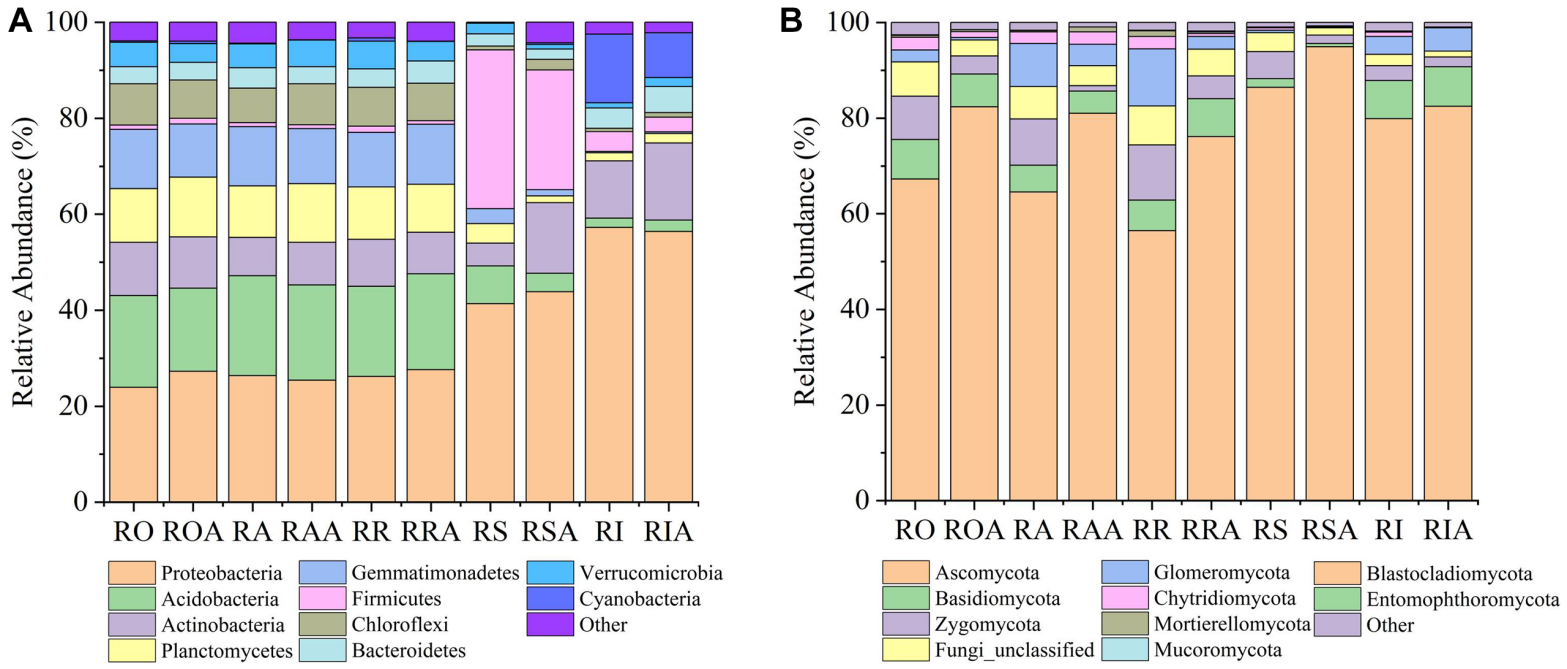
Changes in relative abundance of bacteria and fungi between control and AMF treatment. **(A)** bacteria and **(B)** fungi. RO (bulk soil without AMF treatment); ROA (bulk soil with AMF treatment); RA (ectorhizosphere soil without AMF treatment); RAA (ectorhizosphere soil with AMF treatment); RR (rhizosphere soil without AMF treatment); RRA (rhizosphere soil with AMF treatment); RS (rhizoplane without AMF treatment); RSA (rhizoplane with AMF treatment); RI (endosphere without AMF treatment) and RIA (endosphere with AMF treatment).

In compartments that were closer to the root, which includes RO and RI, we also noticed a considerable rise in the relative abundance of dominating bacteria. For instance, the abundance of several taxa (Proteobacteria), which were enriched in the RI, climbed progressively from RO, RA, RR, and RS to RI. However, from RO, RA, RR, and RS to RI, the relative abundances of some taxa (Planctomycetes, Gemmatimonadetes, and Acidobacteria) dropped. These findings suggest that the enriched taxa are significant endophytes in the RI. Notably, the enrichment of various bacteria in the RIA was impacted by the AMF inoculation. AMF treatment, for instance, led to a gradual increase in the relative abundance of Actinomycetes from RAA to RSA to RIA. Verrucomicrobia and Planctomycetes gathered in the RIA, which contrasts with their abundance in the absence of AMF (Figure 3A).

Following inoculation with AMF, the fungal gradient changed from RO, RA, RR, and RS to RI (Figure 3B). From RAA, RRA, and RSA to RIA, Mortierellomycota’s relative abundance declined. In response to AMF treatment, the trend of Basidiomycota and Ascomycota abundance did not alter, but their relative abundances changed. In response to AMF treatment, the relative abundance of Zygomycota dropped in RAA samples but increased in RIA samples. From RAA to RRA to RSA, Glomeromycota levels dropped along a gradient. These findings suggest that the enrichment or composition of RAA and RRA fungi are impacted by AMF inoculation.

### 3.4 Co-occurrence network analysis of the core microbial community

Heat maps were used to differentiate between taxa with high and low abundances within the top 10 most prevalent genera in order to examine the differential changes in bacterial community structure following AMF injection. Regarding bacterial populations, following AMF inoculation, the peanut rhizosphere had rather high levels of *Sphingomonas* and *Bacillus* at the genus level. Following AMF inoculation, the fungal communities *Talaromyces* and *Ascomycota* at the genus level were relatively prevalent in the peanut rhizosphere. Accordingly, all of the findings demonstrate that AMF causes bacterial and fungal communities to shift and *Talaromyces* and *Ascomycota* to be enriched at the genus level of fungi (Supplementary Figure S6).

**Figure 4:**
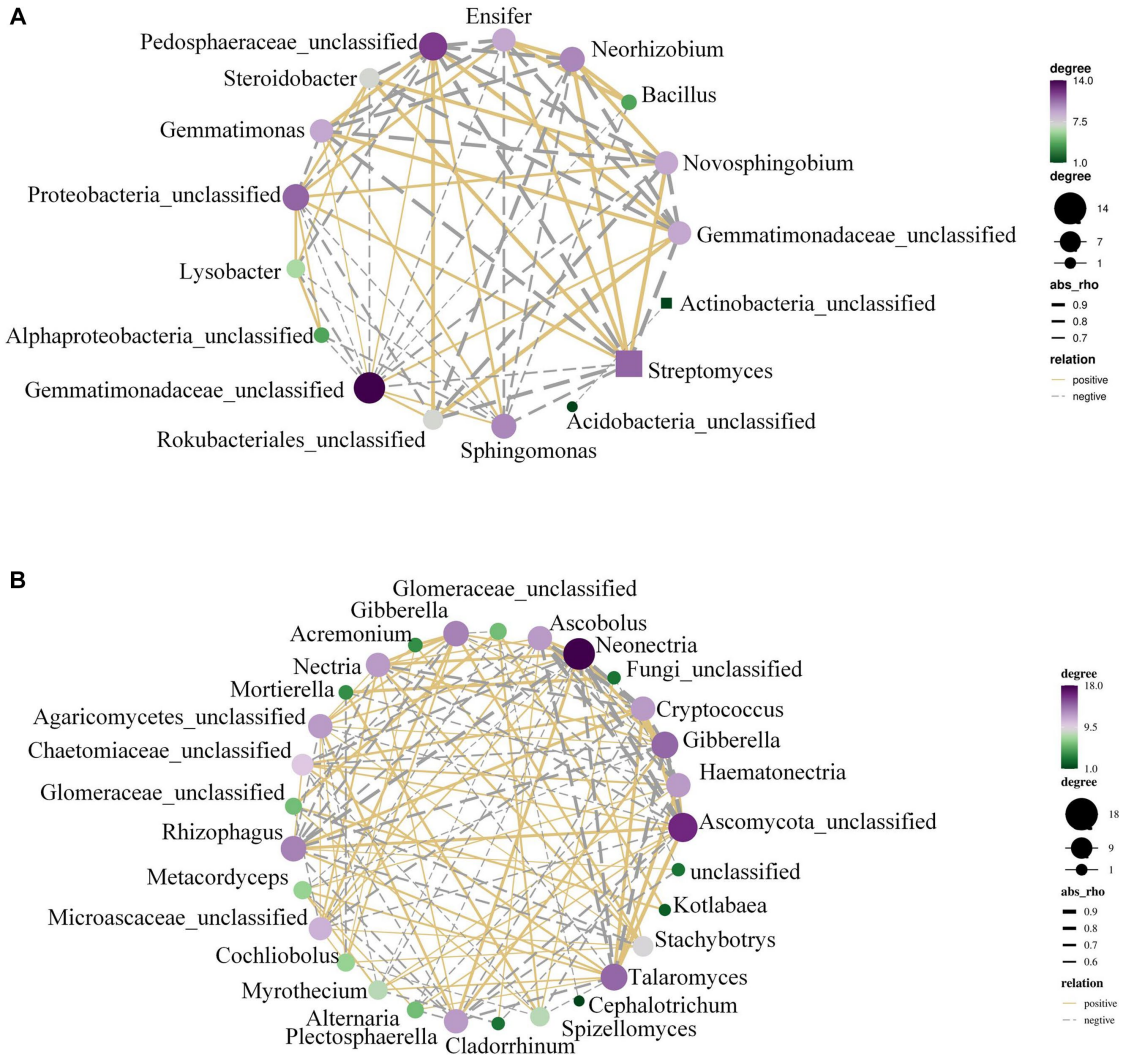
The OTU network of peanut rhizosphere bacteria. **(A)** Control and **(B)** AMF treatment. A connection stands for a strong (Spearman’s r > 0.6) and significant (*p* < 0.01) correlation.

The rhizosphere microbiota in Figure 4A revealed that AMF infestation led to considerable changes in the abundance of microbial communities such *Gemmatimonadetes* and *Actinobacteria*. To further explain this, the microbial co-occurrence networks were examined.

With the help of Supplementary Figure S4B, we conducted a study using combined datasets of bacterial and fungal OTUs to analyze the abundance of microbial communities and co-occurrence networks. It was discovered that *Talaromyces*, *Chaetomiaceae*, *Mortierella*, etc., were the sources of the majority of co-occurrence associations (Figure 4B). Analysis of co-occurrence networks revealed that the AMF treatment was responsible for altering and strengthening the linkages within microbial communities. There was a notable increase in plant-beneficial microbes (such as *Talaromyces* and *Rhizophagus*, etc.). AMF and nitrogen-fixing bacteria exhibit a strong correlation within the soil ecosystem. AMF enhance the plant’s phosphorus absorption capacity through a symbiotic association with the plant’s roots, thereby fostering plant growth. Simultaneously, nitrogen-fixing bacteria provide plants with readily available nitrogen, a vital element for plant development. The cooperative interplay between these two components is crucial for crop production and can significantly affect yield.

### 3.5 Soil enzyme activity

**Figure 5:**
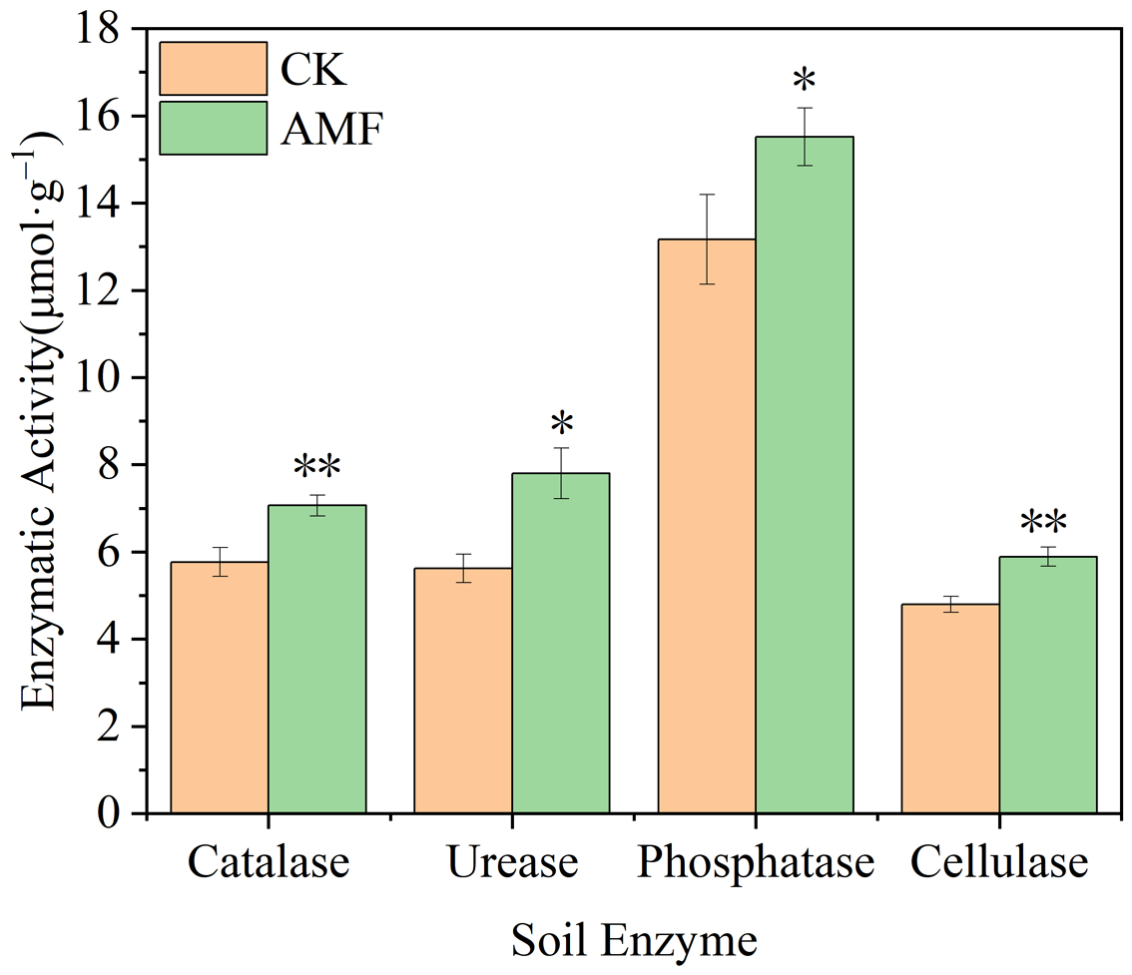
Changes of soil enzyme activity after inoculation with AMF. * and ** represent significance (*p* < 0.05) and high significance (*p* < 0.01).

According to Figure 5, the soil catalase activity increased over time and peaked at 7.06 ± 0.24 μmol·g^−1^ in the AMF treatment, representing an increase of 18.27% compared to control group (*p* < 0.01). The highest urease activity measured was 7.80 ± 0.58 μmol·g^−1^, which was 27.95% greater than control group (*p* < 0.05). The highest activity of phosphatase at the period was 15.52 ± 0.66 μmol·g^−1^, 15.14% greater than that of control group (*p* < 0.05). The greatest activity was 5.89 ± 0.22 μmol·g^−1^, which was 21.00% greater than that of control group (*p* < 0.01) and the highest ever recorded. In this study, the application of AMF boosted the enzyme activities of soil enzymes, indicating that the planting of AMF application can promote soil fertility.

### 3.6 Functional prediction of the bacterial community

**Figure 6:**
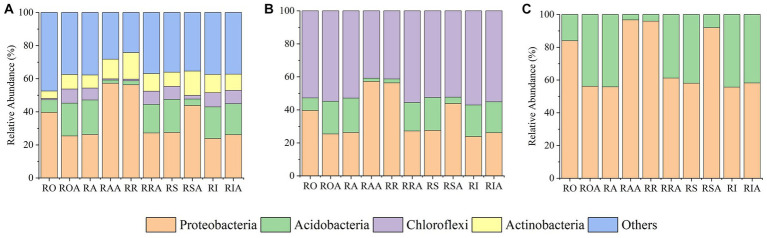
The relative abundance of bacterial phenotype after inoculation with AMF. **(A)** aerobic bacteria, **(B)** anaerobic bacteria, and **(C)** facultative anaerobic bacteria. RO (bulk soil without AMF treatment); ROA (bulk soil with AMF treatment); RA (ectorhizosphere soil without AMF treatment); RAA (ectorhizosphere soil with AMF treatment); RR (rhizosphere soil without AMF treatment); RRA (rhizosphere soil with AMF treatment); RS (rhizoplane without AMF treatment); RSA (rhizoplane with AMF treatment); RI (endosphere without AMF treatment) and RIA (endosphere with AMF treatment).

Using BugBase, a tool that assesses the percentage of bacteria belonging to each of the several phenotypes in a sample, three probable bacterial phenotypes—aerobic, facultative anaerobic, and anaerobic—were projected across various compartments. AMF treatment resulted in significant variations in the proportions of facultative anaerobic and aerobic bacteria (*p* > 0.05), but not in the relative abundance of anaerobic bacteria. Following AMF inoculation, the abundance of aerobic bacteria in the bacterial community showed an increased trend, primarily driven by Actinobacteria (Figure 6A). This pattern was also influenced by the abundance of Acidobacteria, Chloroflexi, and Proteobacteria. Anaerobic bacterial abundance generally indicated a declining trend (Figure 6B), especially for Acidobacteria. Changes in the relative abundance of Proteobacteria was the cause of the rising abundances of facultative anaerobic bacteria (Figure 6C).

The function of peanut rhizosphere microorganisms was predicted, and the results are shown in Supplementary Figure S7. After AMF inoculation, the abundances of several genes, including those for phenylacetate-CoA ligase, taurine-2-oxoglutarate transaminase, (S)-citramalyl-CoA lyase, ceramide glucosyltransferase, and D-cysteine desulfhydrase, were significantly higher (*p* < 0.05). Following AMF inoculation, ITS1 showed a significantly (*p* < 0.05) greater abundance for the gene encoding glucan 1, 3-β-glucosidase, a crucial enzyme that inhibits the growth of pathogenic fungi. Other genes for nucleic acid and carbon metabolism, such as exodeoxyribonuclease I, citrate (Si)-synthase, Cu^2+^-exporting ATPase, glycogenin glucosyltransferase, and sterol 3-glucosyltransferase, were also markedly more abundant after AMF inoculation. However, following AMF inoculation, the abundances of genes encoding long-chain fatty acid-CoA ligase and L-gulonolactone oxidase decreased.

### 3.7 Effect of AMF application on growth performance and yield of peanut

As a result of AMF application, the main stem height and lateral branch length increased (Table 1), while the number of lateral branches and leaves did not change significantly compared to the controls. Additionally, the peanut pod yield in the AMF group showed an upward trend. The 100-pod weight and 100-seed weight of H22 increased by 20.02% and 22.31%, respectively, following AMF inoculation. Moreover, the overall number of pods produced by each plant increased after AMF inoculation, although the difference from the control was not very pronounced. Therefore, AMF application can promote peanut development in saline-alkaline soil and increase pod yield.

**Table 1:** Effects of AMF application the morphology and yield of peanut.

Treatment	Plant morphology			Yield		
	Main stem height (cm)	Lateral branch length (cm)	Main stem leaf number (cm)	100 pods weight (g)	100 seeds weight (g)	The number of total pods
Control group	19.30 ± 0.80	21.20 ± 1.20	4.60 ± 0.30	183.90 ± 15.45	76.00 ± 5.86	13.27 ± 1.14
Experiment group	25.70 ± 1.10**	28.27 ± 0.85**	5.40 ± 0.60	229.93 ± 13.71*	97.83 ± 4.86**	15.50 ± 0.40

* and ** represent significance (*p* < 0.05) and high significance (*p* < 0.01).

## 4 Discussion

The primary threat to global agricultural productivity is soil salinity, which significantly hinders the growth and yield of groundnut crops. Therefore, it is crucial to explore effective strategies for mitigating the adverse effects of saline soil on global peanut production. Studies have shown that rhizosphere-associated bacteria play a vital role in influencing a plant’s ability to tolerate salt stress (Ullah et al., 2017, 2019). To address the detrimental consequences of saline soil on groundnut cultivation, the utilization of AMF emerges as a potentially valuable and efficient approach. In this study, we employed functional prediction and high-throughput sequencing techniques to investigate the characteristics of the microbial community structure in the rhizosphere soil of peanut plants following mycorrhizal inoculation.

AMF inoculation significantly increased the relative abundance of Proteobacteria, Actinobacteriota, Chloroflexi, and Bacteroidetes, which are among the top 10 phyla. Protozoa are abundant, widely distributed, and play a crucial role in agricultural soils (Fierer, 2017). Additionally, Actinobacteria are highly beneficial in decomposing organic matter, making molecules more readily absorbable by plants (Servin et al., 2008). Bacteroidetes are saprophytic organisms responsible for breaking down complex organic compounds (Thomas et al., 2011). In our research, we observed higher densities of Firmicutes in RSA and RIA. Maheshwari et al. (2011) found that various members of the Firmicutes phylum inhabit different agricultural niches, enhancing crop yield through processes such as phytohormone production, antibiotic release, phosphate solubilization, atmospheric N_2_ fixation, NH_3_ release, and other mechanisms. Actinomycetes and Bacteroides have been shown to have high salinity tolerance in previous studies (Zhao et al., 2020; Chi et al., 2021). These changes in the microbial community structure have the potential to enhance the saline-alkali tolerance of the H22 variety and subsequently increase its productivity in saline-alkali soil.

The soil–plant compartment had an impact on the composition of the fungal community. Our research revealed that the AMF groups exhibited lower fungal diversity. Ascomycota and Basidiomycota, which accounted for between 62.00% and 89.00% of the relative abundance in the soil–plant compartments, constituted the majority of the major fungal phyla in our study. In contrast to the root endosphere, Mucoromycota fungi were more commonly found in the rhizosphere and bulk soil. Among these, the Mortierellaceae family, particularly the genus *Mortierella*, was dominant. According to our findings, the *Mortierella* accounted for 6.14% of the relative abundance of all genera, making it one of the most prevalent genera. Notably, Mortierella plays a significant role in phosphorus cycling in the rhizosphere (Osorio and Habte, 2001). One significant AMF genus that influences plants is known as Glomus (Landwehr et al., 2002). Previous research has demonstrated that inoculation with Glomus promotes plant growth and biomass (Campos-Soriano et al., 2010; Naseer et al., 2022). In our study, *Glomus* was the most prevalent AMF genus in both the CK and AMF groups (Figure 5). AMF offers a wide range of advantages and potential value in harsh environments (Guo et al., 2022). According to a recent study by Salvioli et al. (2016), bacteria associated with AMF enhance AMF fitness and soil nutrient uptake. Estrada et al. (2013) demonstrated how mycorrhizal inoculation improved potassium accumulation and reduced sodium ion levels in plants experiencing salt stress. These findings collectively suggest that AMF inoculation may enhance the nitrogen and phosphorus cycles and promote the development of H22 in saline-alkaline soil.

Soil enzymes, primarily produced by microorganisms, play vital roles in organic material decomposition and ecosystem processes. In saline-alkaline soil areas, key enzymes like catalase, urease, phosphatase, sucrase, and cellulase are used as indicators to predict soil ecosystem function and environmental quality (Qu et al., 2020). Catalase, for example, breaks down hydrogen peroxide and enhances soil oxidation (Guo et al., 2015). Urease activity indicates potential changes in soil nitrogen content (Jia et al., 2017). Phosphatase generates phosphate ions, contributing to soil nutrient availability. Sucrase hydrolyzes sucrose, improving soil nutrients availability. AMF enhances plant root nitrogen uptake by boosting soil enzyme activity in the rhizosphere (Liu and Chen, 2021). Soil dehydrogenase activity, representing soil microbial metabolism, can reflect microbial redox capacity (Xu et al., 2016). Soil phosphatase, which catalyzes the mineralization of soil organic phosphorus, marginally increased following AMF inoculation (Hu et al., 2014). AMF, by chelating trace elements, can alter the macro-to-trace element ratio in the soil. This promotes the production of sugars, minerals, lipids, and various vitamins through biosynthesis. AMF also enhances microbial mineralization in the soil, particularly the breakdown of polysaccharides into soluble monomers. Consequently, cultivating H22 and applying AMF inoculation on saline-alkaline lands have significant potential to improve soil properties, a critical task for increasing crop production capacity and preserving soil fertility. These empirical findings have been unequivocally confirmed through yield assessments, demonstrating that the implementation of AMF inoculation can substantially increase H22 yields on saline-alkaline terrain.

The majority of soil metabolites come from microbes and plant roots. Depending on the plant species, genotype, and environmental factors, different root exudates have different compositions. Our functional prediction suggests that AMF encourage various microbial enzyme creations, which could quicken soil metabolism. Furthermore, based on a functional prediction (Supplementary Figure S7B), the activities of the dioxygenases flavanone 3-dioxygenase and hydroxyquinol 1, 2-dioxygenase increased in the presence of AMF. These enzymes efficiently degrade aromatic compounds by initiating the breakage of aromatic rings and hydroxylating the benzene rings of aromatic compounds with the help of molecular oxygen.

AMF can thereby expedite the decomposition of aromatic chemicals. According to prior research (Vacheron et al., 2013), these aromatic chemicals may induce chemotactic responses in soil microorganisms, particularly plant growth-promoting rhizobacteria (PGPR). AMF therapy thereby affects the microbial community’s structure and function, as well as the host’s ability to attract PGPRs, consequently promoting H22 expansion.

## 5 Conclusion

This study highlights how AMF can enhance the yield of salt-sensitive plants in saline-alkali conditions. After AMF application, there was a positive shift in the soil microbial community, favoring aerobic and facultative anaerobic bacteria over anaerobic species. Additionally, AMF significantly increased soil enzyme activity, particularly those inhibiting pathogens and aiding organic carbon degradation. It also improved metabolic pathways related to amino acids and carbohydrates, mineralization, and reduced enzymes involved in fatty acid biosynthesis, while boosting pathogen-inhibiting enzymes in the rhizosphere. In saline-alkaline soil, AMF led to a remarkable 20.02% increase in 100-fruit weight and a 22.30% rise in 100-grain weight compared to the control. This improvement is attributed to the creation of a more diverse microenvironment and heightened enzyme activity facilitated by AMF.

Importantly, AMF played a vital role in shaping the microbial population structure around peanut roots, creating an environment conducive to plant growth. These findings offer valuable insights into how AMF enhance salt resistance and overall health of peanut plants in saline-alkaline soil.

## Data availability statement

The datasets presented in this study are deposited in the NCBI database (https://www.ncbi.nlm.nih.gov/bioproject/) under accession number PRJNA1023039.

## Author contributions

JK: Writing – original draft, Writing – review & editing. WY: Writing – original draft, Writing – review & editing. SL: Writing – review & editing. NX: Writing – review & editing. YS: Writing – review & editing. YG: Writing – review & editing. MA: Writing – review & editing. YZ: Writing – review & editing. SY: Writing – review & editing. RM: Writing – review & editing. GC: Funding acquisition, Project administration, Writing – review & editing.
