# Supplementary material for: Arbuscular mycorrhizal fungi increased peanut (Arachis hypogaea L.) yield by changing the rhizosphere microbial community structure in saline-alkali soil
Source: Front Microbiol. 2023 Dec 8;14:1303979. doi: 10.3389/fmicb.2023.1303979 (PMC10748501; doi:10.3389/fmicb.2023.1303979)
Supplement: Supplementary file 1 [file Data_Sheet_1.docx]

Supplementary Material

# Supplementary Figures and Tables

## Supplementary Figures

##
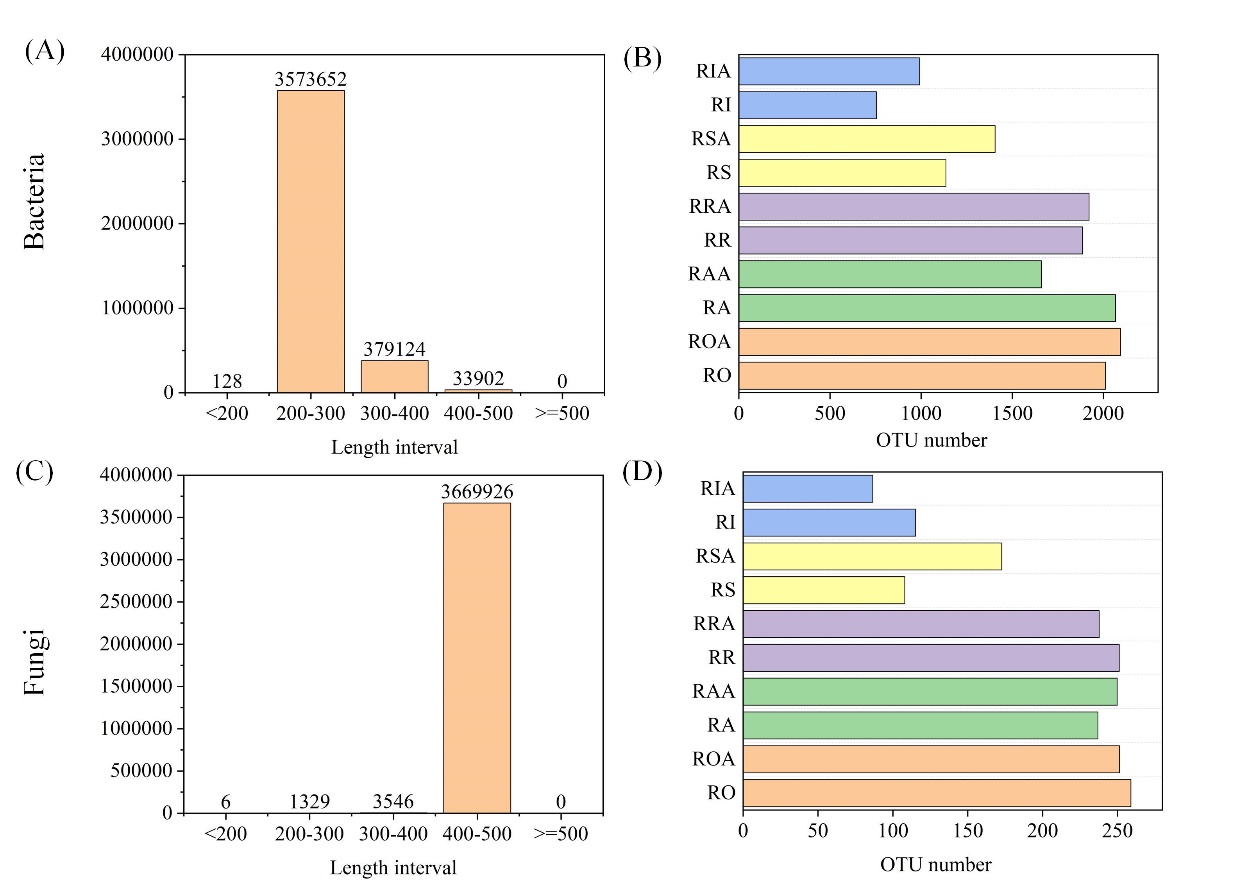


**Supplementary Figure 1.** Overall sequence data and alpha diversity analysis. **(A)** The sequence length of bacteria. **(B)** Operational taxonomic units (OTUs) of bacteria of difference soil groups. **(C)** The sequence length of fungi. (D) Operational taxonomic units (OTUs) of fungi of difference soil groups. RO (bulk soil without AMF treatment); ROA (bulk soil with AMF treatment); RA (ectorhizosphere soil without AMF treatment); RAA (ectorhizosphere soil with AMF treatment); RR (rhizosphere soil without AMF treatment); RRA (rhizosphere soil with AMF treatment); RS (rhizoplane without AMF treatment); RSA (rhizoplane with AMF treatment); RI (endosphere without AMF treatment) and RIA (endosphere with AMF treatment).


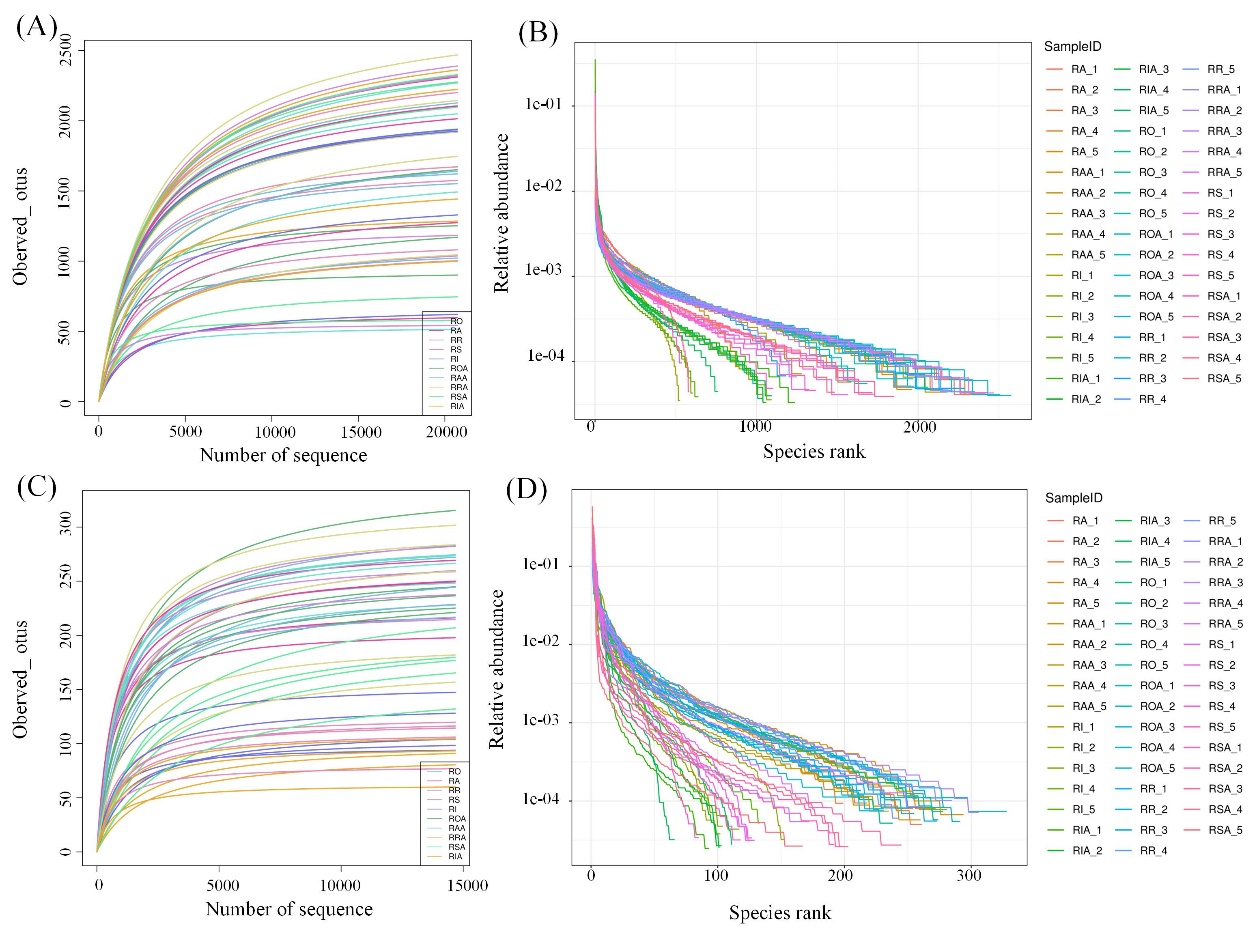


**Supplementary Figure 2.** Rarefaction curve and rank abundance curve of 16S rRNA gene and ITS1 gene sequencing. **(A)** Rarefaction curve analysis showing the depth of 16S rRNA gene sequencing and the possibility of diversity. **(B)** Rank abundance curve showing the relative bacteria species abundance and evenness. The length of the polyline on the horizontal axis reflects the OTU numbers and represents the richness of the bacterial community. The flatness of the polyline reflects the evenness of the bacterial community composition. **(C)** Rarefaction curve analysis showing the depth of ITS1 gene sequencing and the possibility of diversity. **(D)** Rank abundance curve showing the relative bacteria species abundance and evenness. The length of the polyline on the horizontal axis reflects the OTU numbers and represents the richness of the bacterial community. The flatness of the polyline reflects the evenness of the bacterial community composition. RO (bulk soil without AMF treatment); ROA (bulk soil with AMF treatment); RA (ectorhizosphere soil without AMF treatment); RAA (ectorhizosphere soil with AMF treatment); RR (rhizosphere soil without AMF treatment); RRA (rhizosphere soil with AMF treatment); RS (rhizoplane without AMF treatment); RSA (rhizoplane with AMF treatment); RI (endosphere without AMF treatment) and RIA (endosphere with AMF treatment).


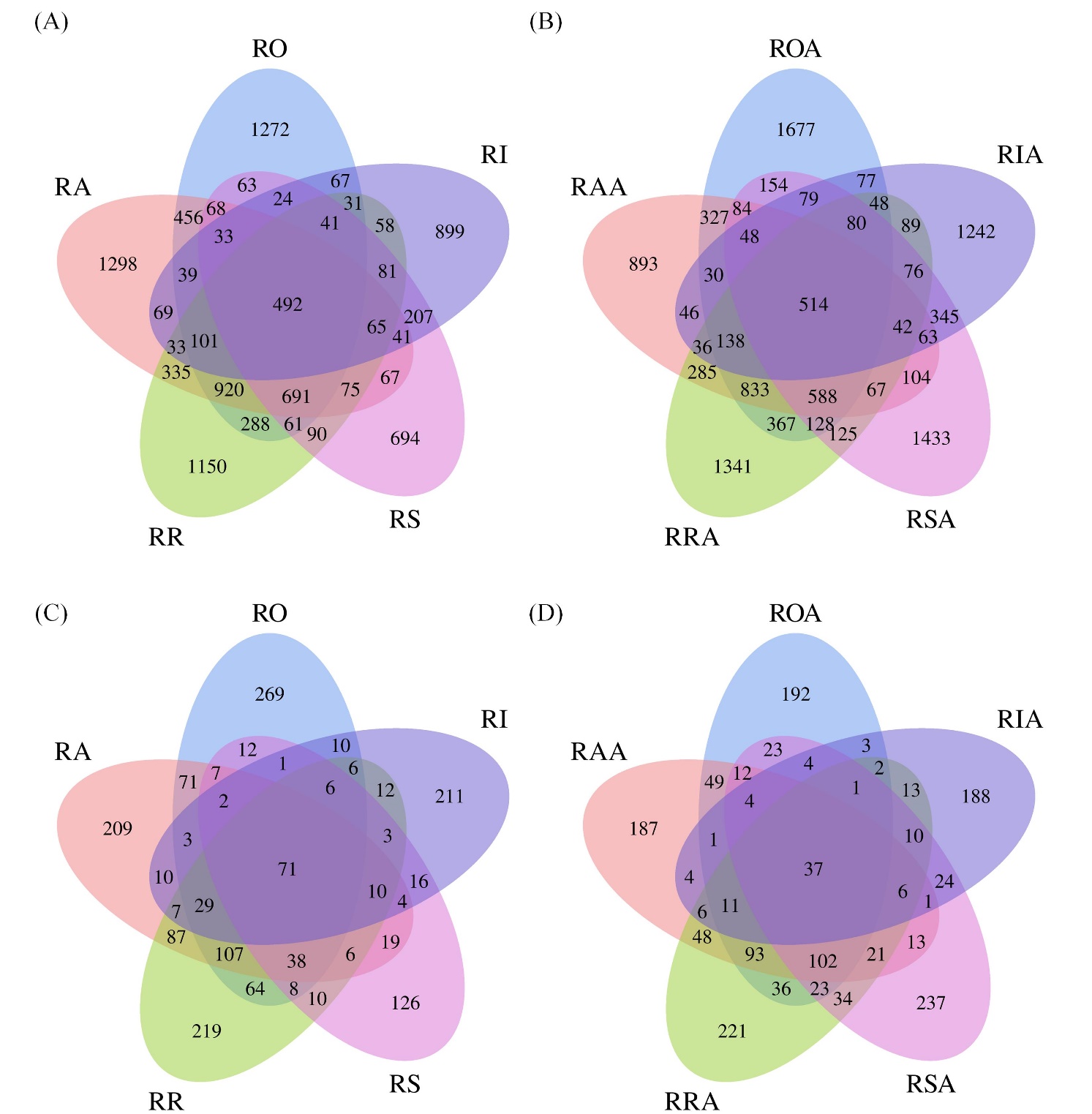


**Supplementary Figure 3.** Variation of bacteria and fungi between control and AMF inoculation. **(A)** 16S rRNA gene without AMF inoculation, **(B)** 16S rRNA gene with AMF inoculation, **(C)** ITS without AMF inoculation and **(D)** ITS with AMF inoculation. RO (bulk soil without AMF treatment); ROA (bulk soil with AMF treatment); RA (ectorhizosphere soil without AMF treatment); RAA (ectorhizosphere soil with AMF treatment); RR (rhizosphere soil without AMF treatment); RRA (rhizosphere soil with AMF treatment); RS (rhizoplane without AMF treatment); RSA (rhizoplane with AMF treatment); RI (endosphere without AMF treatment) and RIA (endosphere with AMF treatment).


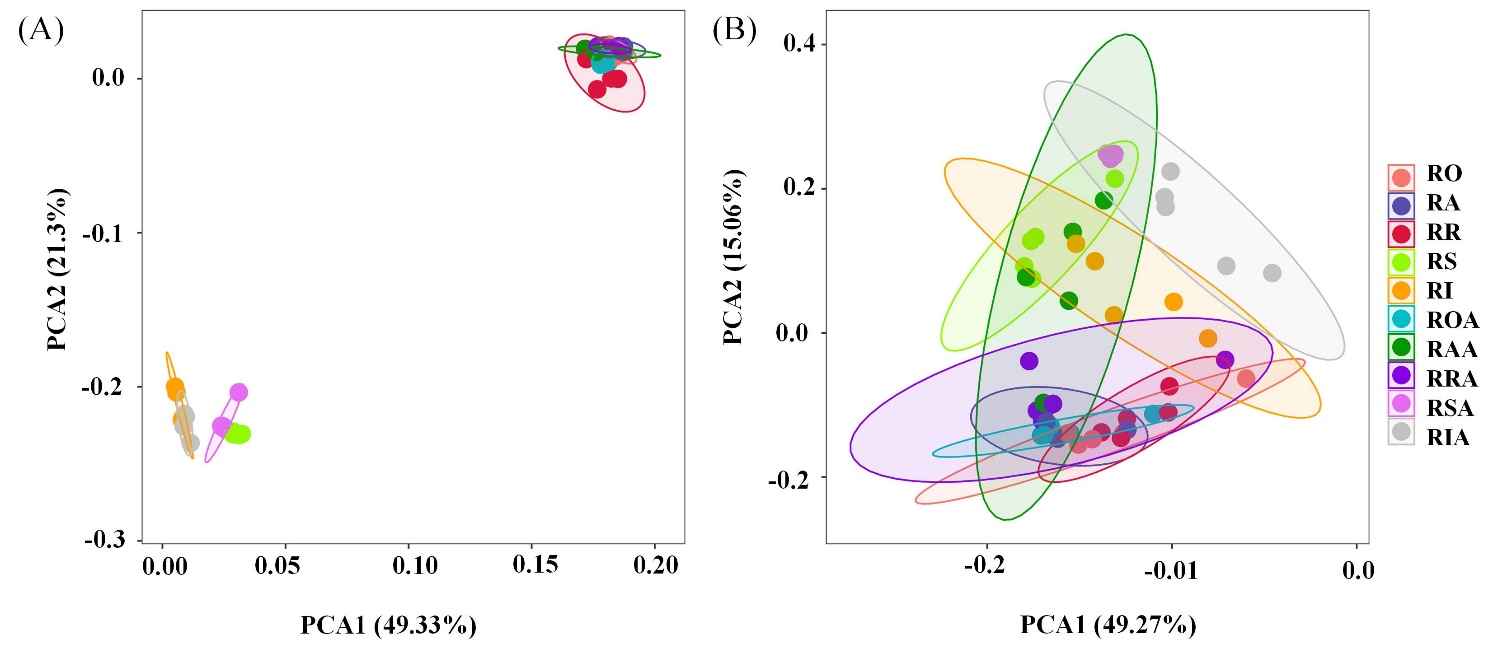


**Supplementary Figure 4**. Principal component analysis (PCA) of communities based on 16S rRNA gene and ITS gene sequences. **(A)** Bacterial, **(B)** fungal. RO (bulk soil without AMF treatment); ROA (bulk soil with AMF treatment); RA (ectorhizosphere soil without AMF treatment); RAA (ectorhizosphere soil with AMF treatment); RR (rhizosphere soil without AMF treatment); RRA (rhizosphere soil with AMF treatment); RS (rhizoplane without AMF treatment); RSA (rhizoplane with AMF treatment); RI (endosphere without AMF treatment) and RIA (endosphere with AMF treatment).

**
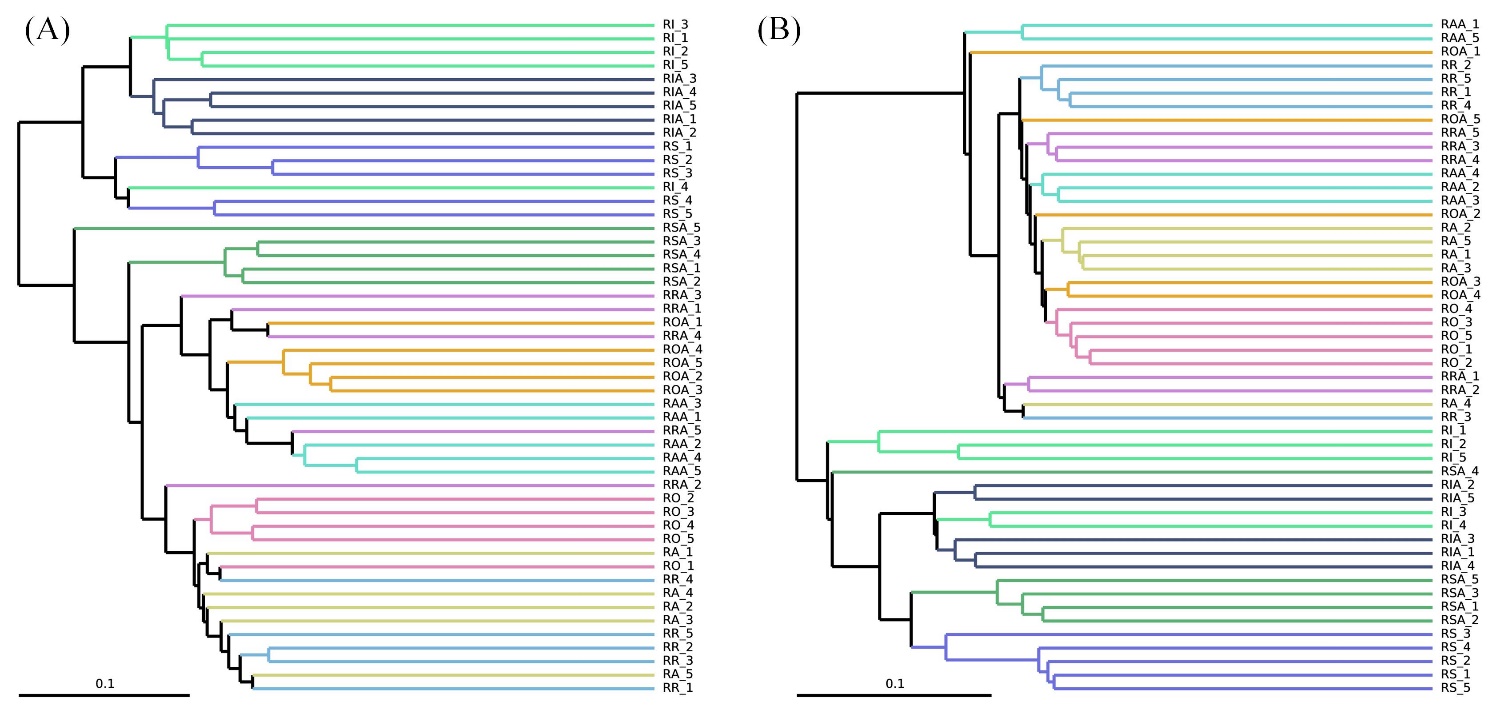
Supplementary Figure 5.** Unweighted pair group method with arithmetic mean (UPGMA) analysis. The UPGMA analysis showing the degree of similarity of different soil bacteria **(A)** and fungi **(B)** community structures, which is clustered according to the similarity of their components in different soil groups. RO (bulk soil without AMF treatment); ROA (bulk soil with AMF treatment); RA (ectorhizosphere soil without AMF treatment); RAA (ectorhizosphere soil with AMF treatment); RR (rhizosphere soil without AMF treatment); RRA (rhizosphere soil with AMF treatment); RS (rhizoplane without AMF treatment); RSA (rhizoplane with AMF treatment); RI (endosphere without AMF treatment) and RIA (endosphere with AMF treatment).


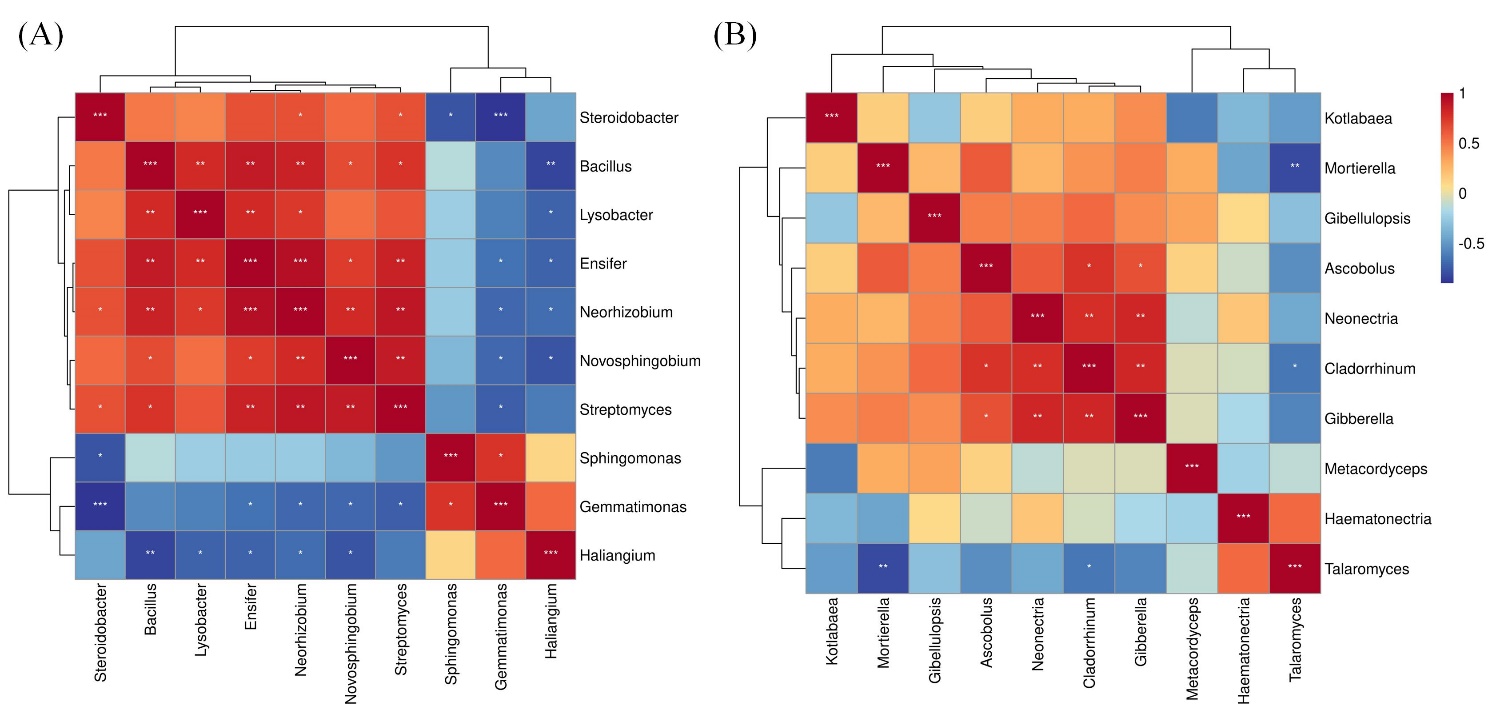


**Supplementary Figure 6.** Correlation of the top 10 bacteria and fungi. (A) Correlation of the top 10 bacteria based on relative abundance. (B) Correlation of the top 10 fungi based on relative abundance. Red and blue indicate positive and negative correlation, respectively. *, ** and *** represent significance (*p* < 0.05), high significance (*p* < 0.01) and (*p* < 0.001).


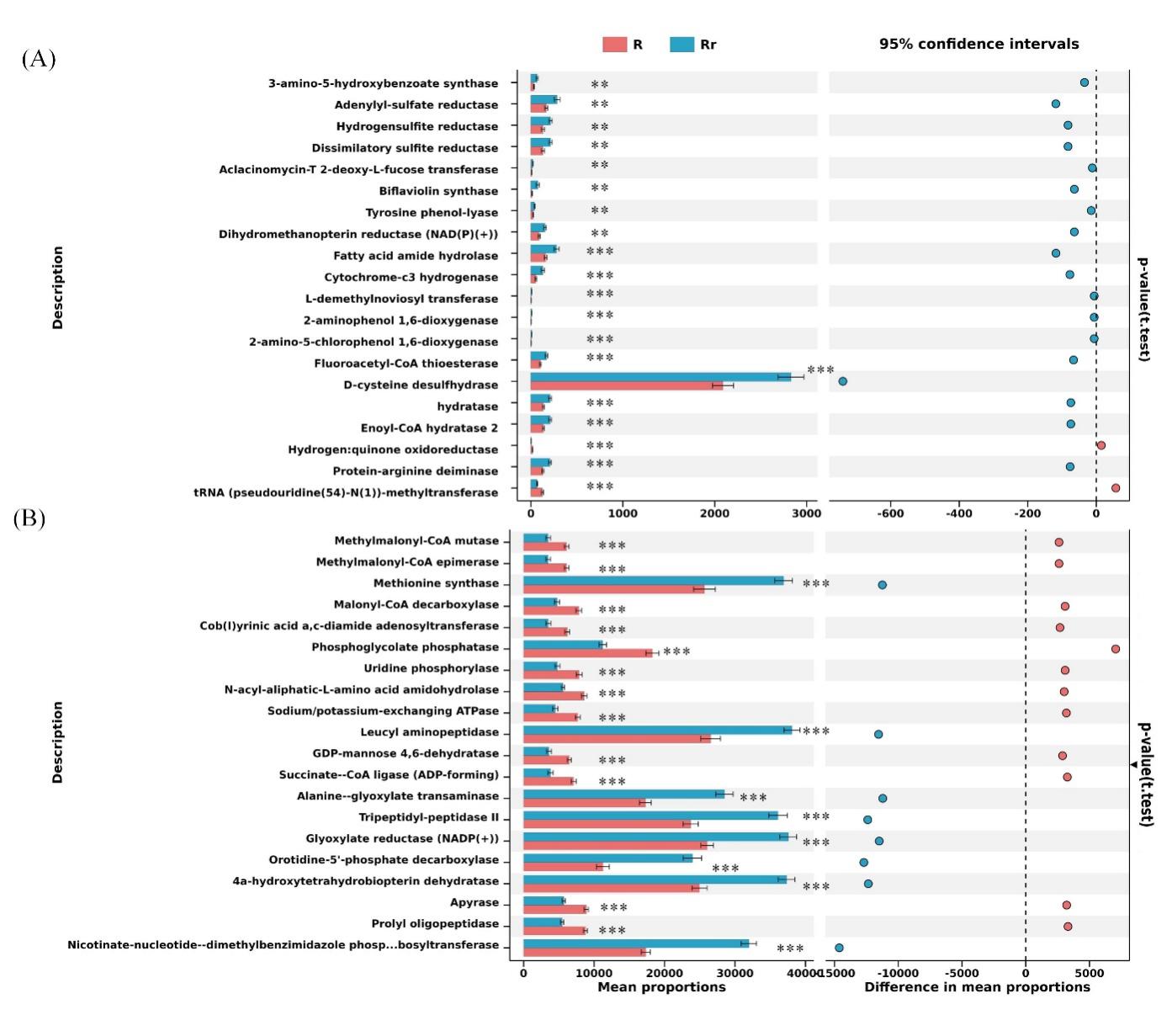


**Supplementary Figure 7.** Functional prediction based on PICRUSt2. R: control group; Rr: treatment group .16S rRNA gene (A) and ITS (B). ** and *** represent significance high significance (*p* < 0.01) and (*p* < 0.001).

## Supplementary Tables

**Supplementary Table 1.** Top 10 phylums of bacteria at the phylum level.

| Phylum | Control group (%) | Experimental group (%) |
| --- | --- | --- |
| Proteobacteria | 34.65 | 36.08 |
| Acidobacteria | 13.62 | 12.65 |
| Actinobacteria | 9.06 | 11.79 |
| Planctomycetes | 7.66 | 7.60 |
| Gemmatimonadetes | 7.84 | 7.33 |
| Firmicutes | 7.79 | 6.14 |
| Chloroflexi | 5.06 | 5.49 |
| Bacteroidetes | 3.68 | 3.89 |
| Verrucomicrobia | 3.78 | 3.26 |
| Cyanobacteria | 3.14 | 2.07 |

**Supplementary Table 2.** Top 10 genus of bacteria at the genus level.

| Genus | Control group (%) | Experimental group (%) |
| --- | --- | --- |
| *Bacillus* | 6.76 | 5.20 |
| *Sphingomonas* | 2.04 | 1.43 |
| *Gemmatimonas* | 1.30 | 1.01 |
| *Neorhizobium* | 1.08 | 0.93 |
| *Novosphingobium* | 1.04 | 0.89 |
| *Ensifer* | 0.94 | 0.83 |
| *Steroidobacter* | 0.76 | 0.84 |
| *Haliangium* | 0.64 | 0.94 |
| *Lysobacter* | 1.36 | 1.21 |
| *Streptomyces* | 0.72 | 1.76 |

**Supplementary Table 3.** Top 10 phylums of fungi at the phylum level.

| Phylum | Control group (%) | Experimental group (%) |
| --- | --- | --- |
| Ascomycota | 70.92 | 83.39 |
| Basidiomycota | 6.01 | 5.68 |
| Zygomycota | 7.79 | 2.68 |
| Fungi_unclassified | 5.70 | 3.17 |
| Glomeromycota | 5.54 | 2.50 |
| Chytridiomycota | 1.83 | 0.96 |
| Mortierellomycota | 0.41 | 0.40 |
| Mucoromycota | 0.06 | 0.01 |
| Blastocladiomycota | 0.01 | 0.03 |
| Entomophthoromycota | 0.01 | 0.01 |

**Supplementary Table 4.** Top 10 genus of fungi at the genus level.

| Genus | Control group (%) | Experimental group (%) |
| --- | --- | --- |
| *Haematonectria*  *Talaromyces* | 14.15  8.40 | 15.63  14.42 |
| *Gibellulopsis* | 4.89 | 4.82 |
| *Mortierella* | 6.14 | 2.44 |
| *Kotlabaea* | 1.50 | 5.15 |
| *Ascobolus* | 3.39 | 3.24 |
| *Metacordyceps* | 4.66 | 1.84 |
| *Neonectria* | 2.09 | 3.16 |
| *Gibberella* | 2.52 | 1.97 |
| *Cladorrhinum* | 2.20 | 1.13 |
